# Supplementary material for: Larval exposure to field-realistic concentrations of clothianidin has no effect on development rate, over-winter survival or adult metabolic rate in a solitary bee, Osmia bicornis
Source: PeerJ. 2017 Jun 20;5:e3417. doi: 10.7717/peerj.3417 (PMC5480390; doi:10.7717/peerj.3417)
Supplement: Table S1 — Method detection limits (MDLs), method quantification (MQLs) limits and absolute recoveries (n = 4) of five neonicotinoids, for pollen samples extracted using the QuEChERS method and analysed by UHPLC-MS/MS. TMX, thiamethoxam; CLO, clothianidin; IMC, imidacloprid; ACT, acetamiprid and THC, thiacloprid. [file peerj-05-3417-s001.pdf]

Chemical	MDL	MQL	Recovery (*1.2 ppb ww*)	
	ng/g ww		*Mean %*	*SD*
**TMX**	0.12	0.36	95	3
**CLO**	0.12	0.36	93	14
**IMC**	0.16	0.48	92	18
**ACT**	0.04	0.12	83	15
**THC**	0.04	0.12	93	8
