# Supplementary material for: Larval exposure to field-realistic concentrations of clothianidin has no effect on development rate, over-winter survival or adult metabolic rate in a solitary bee, Osmia bicornis
Source: PeerJ. 2017 Jun 20;5:e3417. doi: 10.7717/peerj.3417 (PMC5480390; doi:10.7717/peerj.3417)
Supplement: Table S2 — Mean (±SD) weight of pollen provisions, larvae, pupae and emerged adults across clothianidin treatments. [file peerj-05-3417-s002.pdf]

| CLO (ppb) | Sex | Provision Weight (mg) | Larval Weight (mg) | Pupal Weight (mg) | Adult Weight (mg) |
|---|---|---|---|---|---|
| 0 | Female | 335.54 ±49.99 | 150.86 ±14.68 | 129.32 ±20.34 | 68.57 ±14.93 |
| 1 | | 337.84 ±44.80 | 162.77 ±24.70 | 139.78 ±18.59 | 79.55 ±10.74 |
| 3 | | 334.45 ±77.15 | 152.09 ±32.20 | 131.87 ±28.67 | 73.81 ±16.85 |
| 10 | | 324.79 ±58.38 | 152.69 ±26.79 | 129.19 ±23.01 | 72.92 ±12.50 |
| 0 | Male | 205.95 ±26.17 | 101.14 ±14.08 | 89.59 ±14.31 | 47.45 ±7.69 |
| 1 | | 239.69 ±39.60 | 112.41 ±23.24 | 94.22 ±11.84 | 47.79 ±9.65 |
| 3 | | 225.61 ±25.57 | 109.21 ±16.55 | 94.58 ±13.86 | 47.69 ±8.98 |
| 10 | | 218.55 ±18.90 | 99.41 ±20.34 | 88.56 ±16.22 | 45.97 ±7.65 |
