# Supplementary material for: Larval exposure to field-realistic concentrations of clothianidin has no effect on development rate, over-winter survival or adult metabolic rate in a solitary bee, Osmia bicornis
Source: PeerJ. 2017 Jun 20;5:e3417. doi: 10.7717/peerj.3417 (PMC5480390; doi:10.7717/peerj.3417)
Supplement: Table S3 — Number of days to emergence for male and female bees across clothianidin treatments. [file peerj-05-3417-s003.pdf]

**CLO (ppb)**	**Male**		**Female**	
	Mean ±SE	*Range*	*Mean ±SE*	*Range*
**0**	7.00 ±1.34	4-14	14.85 ±1.01	6-24
**1**	6.21 ±1.01	2-13	15.83 ±1.10	7-26
**3**	5.93 ±0.98	1-10	15.06 ±0.95	7-24
**10**	5.27 ±0.98	1-11	16.34 ±1.01	8-24
