# Supplementary material for: Larval exposure to field-realistic concentrations of clothianidin has no effect on development rate, over-winter survival or adult metabolic rate in a solitary bee, Osmia bicornis
Source: PeerJ. 2017 Jun 20;5:e3417. doi: 10.7717/peerj.3417 (PMC5480390; doi:10.7717/peerj.3417)
Supplement: Table S4 [file peerj-05-3417-s004.pdf]

**Treatment (ppb CLO)**	**Continuous**		**Discontinuous**	
	Male	Female	Male	Female
**0**	1	6	8	8
**1**	3	3	9	8
**3**	2	6	12	10
**10**	1	2	12	11
**TOTAL**	**7**	**17**	**41**	**37**
