# Supplementary figures and images for: Larval exposure to field-realistic concentrations of clothianidin has no effect on development rate, over-winter survival or adult metabolic rate in a solitary bee, Osmia bicornis
Source: PeerJ. 2017 Jun 20;5:e3417. doi: 10.7717/peerj.3417 (PMC5480390; doi:10.7717/peerj.3417)

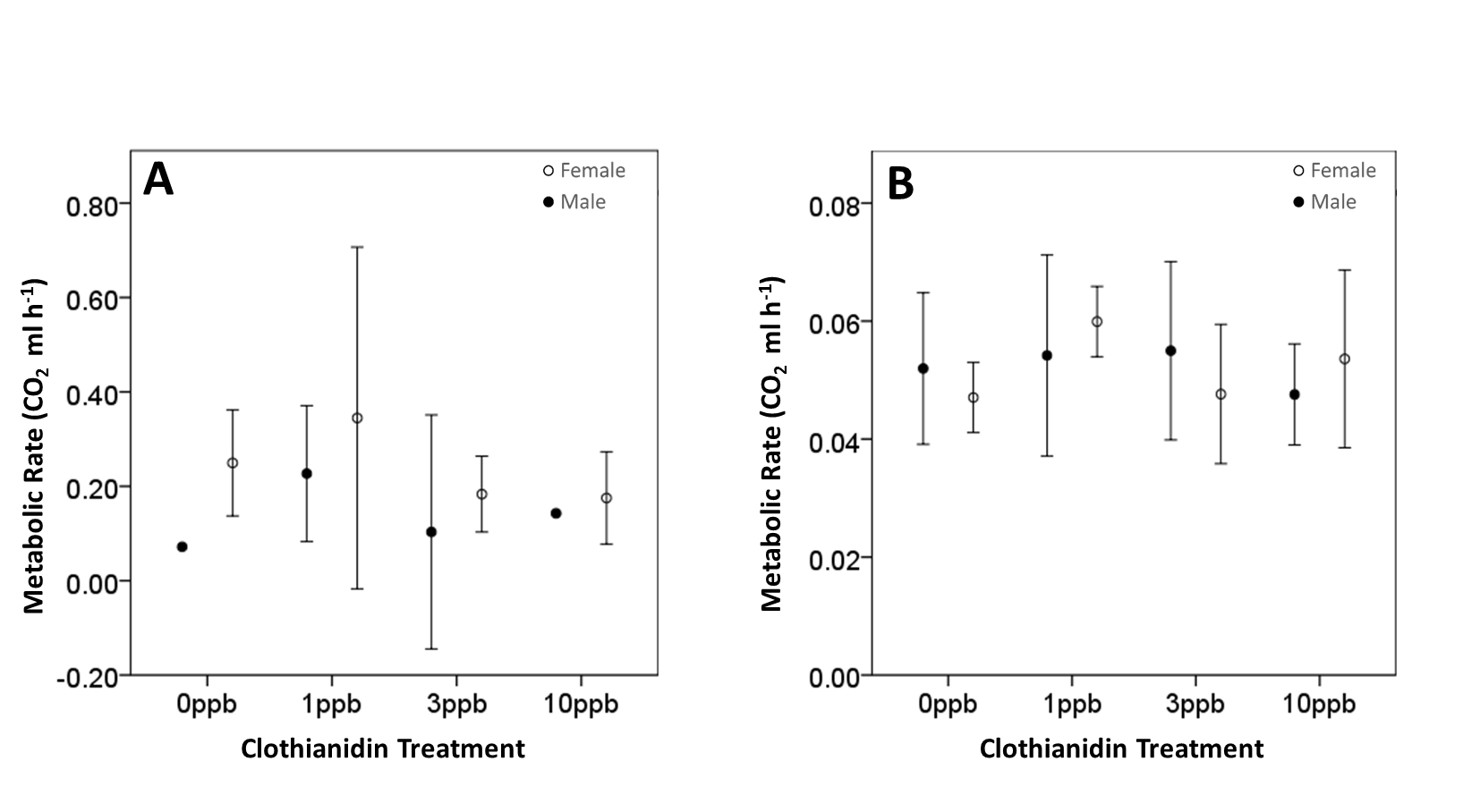

Supplement: Figure S1 — Metabolic rate of bees engaging in (A) continuous (male = black circles n = 7, female = open circles n = 17) and (B) discontinous gas exchange (male = black circles n = 41, female = open circles n = 37). Bees had been exposed to varying concentrations of clothianidin during treatment. Data are means ( ± CI). [file peerj-05-3417-s005.png]
